# [Retracted] Reduced miR‑202 levels enhanced oral cancer development via targeting Sp1

**DOI:** 10.3892/etm.2024.12539

**Published:** 2024-04-15

**Authors:** Jia Zhao, Deguang Ding, Ge Zhao

Exp Ther Med 18:489–496, 2019; DOI: 10.3892/etm.2019.7603

Following the publication of this paper, it was drawn to the Editors’ attention by a concerned reader that certain of the Transwell cell migration and invasion assay data shown in Fig. 3C on p. 492, and the western blotting data shown in Fig. 4E on p. 493 were strikingly similar to data appearing in different form in other articles written by different authors at different research institutes that had already been published elsewhere prior to the submission of this paper to *Experimental and Therapeutic Medicine*. In view of the fact that the abovementioned data had already apparently been published previously, the Editor of *Experimental and Therapeutic Medicine* has decided that this paper should be retracted from the Journal. After having been in contact with the authors, they agreed with the decision to retract the paper. The Editor apologizes to the readership for any inconvenience caused.

